# Hereditary hemorrhagic telangiectasia: genetics and molecular diagnostics in a new era

**DOI:** 10.3389/fgene.2015.00001

**Published:** 2015-01-26

**Authors:** Jamie McDonald, Whitney Wooderchak-Donahue, Chad VanSant Webb, Kevin Whitehead, David A. Stevenson, Pinar Bayrak-Toydemir

**Affiliations:** ^1^Department of Radiology, Hereditary Hemorrhagic Telangiectasia Center, University of UtahSalt Lake City, UT, USA; ^2^Department of Pathology, University of UtahSalt Lake City, UT, USA; ^3^ARUP Institute for Clinical and Experimental PathologySalt Lake City, UT, USA; ^4^Division of Cardiovascular Medicine, Department of Medicine, University of UtahSalt Lake City, UT, USA; ^5^Program in Molecular Medicine, University of UtahSalt Lake City, UT, USA; ^6^George E. Wahlen Veterans Affairs Medical CenterSalt Lake City, UT, USA; ^7^Division of Medical Genetics, Department of Pediatrics, University of UtahSalt Lake City, UT, USA

**Keywords:** HHT, molecular diagnostics, genetics, telangiectasia, arteriovenous malformation, Rendu-Osler-Weber

## Abstract

Hereditary hemorrhagic telangiectasia (HHT) is a vascular dysplasia characterized by telangiectases and arteriovenous malformations (AVMs) in particular locations described in consensus clinical diagnostic criteria published in 2000. Two genes in the transforming growth factor-beta (TGF-β) signaling pathway, *ENG* and *ACVRL1*, were discovered almost two decades ago, and mutations in these genes have been reported to cause up to 85% of HHT. In our experience, approximately 96% of individuals with HHT have a mutation in these two genes, when published (Curaçao) diagnostic criteria for HHT are strictly applied. More recently, two additional genes in the same pathway,* SMAD4* and *GDF2*, have been identified in a much smaller number of patients with a similar or overlapping phenotype to HHT. Yet families still exist with compelling evidence of a hereditary telangiectasia disorder, but no identifiable mutation in a known gene. Recent availability of whole exome and genome testing has created new opportunities to facilitate gene discovery, identify genetic modifiers to explain clinical variability, and potentially define an increased spectrum of hereditary telangiectasia disorders. An expanded approach to molecular diagnostics for inherited telangiectasia disorders that incorporates a multi-gene next generation sequencing (NGS) HHT panel is proposed.

## INTRODUCTION

Hereditary hemorrhagic telangiectasia (HHT) or Rendu-Osler-Weber syndrome is an autosomal-dominantly inherited vascular malformation syndrome characterized by telangiectases and arteriovenous malformations (AVMs) that occurs in 1 in 10,000 individuals (Marchuk et al., 1998). Hallmark features are recurrent epistaxis due to telangiectases of the nasal mucosa; dermal, oral, and gastrointestinal telangiectases; solid-organ AVMs, particularly of the lungs, liver, and brain; and a family history of the same. Presentation with three of these criteria is considered diagnostic for HHT (Shovlin et al., 2000). The dermal telangiectases are generally pinpoint to pinhead sized, very specifically concentrated on the hands, face, and lips, and not diffuse. A typical adult with HHT might have a dozen oral/dermal telangiectases spread between these locations, usually observed only by focused examination. Cutaneous lesions on the limbs and trunk are not characteristic. Epistaxis most often becomes gradually worse, is sometimes stable for long periods of time, but it is essentially unreported for it to spontaneously resolve once started.

Hereditary hemorrhagic telangiectasia exhibits age-related penetrance and the average age of onset varies with manifestation. Approximately 50% of affected individuals have nosebleeds by age ten years and 80–90% by age 21 years. At least 95% eventually develop recurrent nosebleeds. But severity varies tremendously and includes nosebleeds from 3 to 6 times per year of less than a minute in duration, to multiple gushing nosebleeds daily. The percentage of individuals with telangiectases of the hands, lips, face, and oral cavity approaches 100% by later adulthood, but are often not apparent until the second or third decade of life (Plauchu et al., 1989 and Porteous et al., 1992). Cerebral and pulmonary AVMs, however, are largely congenital lesions (Morgan et al., 2002; McDonald et al., 2011b). Significant intra-familial as well as inter-familial variability is the rule; both in terms of the site and number of telangiectases or AVMs, and severity of related symptoms (McDonald et al., 2011b).

In the last two decades multiple genes associated with HHT have been identified and molecular diagnostics has become a routine aspect of medical management, genetic counseling and risk assessment in families with this disorder. This review will summarize current knowledge regarding the genetics of HHT, the phenotypes in molecular subgroups of patients, and the status of molecular diagnostics for HHT. Implications of recent discoveries in genetics and advancements in molecular diagnosis on the clinical definition and diagnosis of HHT will be discussed.

## KNOWN GENES AND PATHWAYS

**FIGURE 1:**
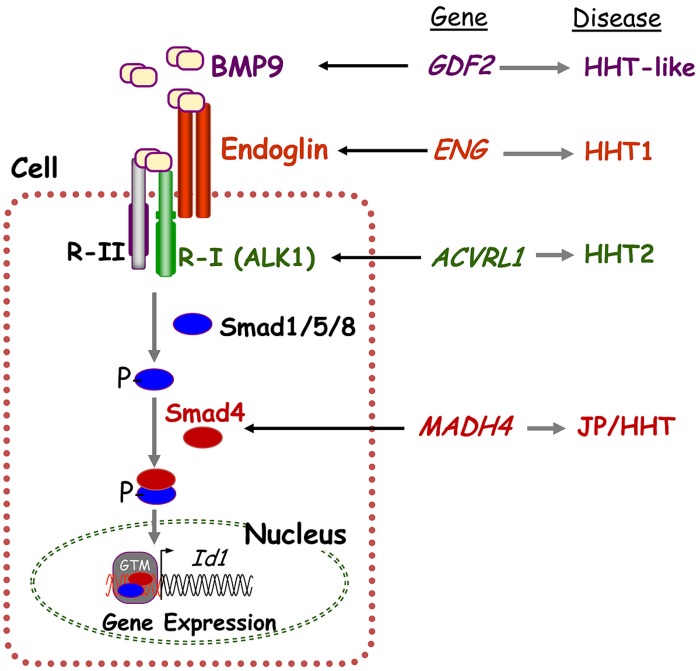
**Hereditary hemorrhagic telangiectasia is a genetically heterogeneous disorder caused by mutations in several genes in the TGF-β/BMP signaling pathway.** BMP9 binds to specific type I (R-I; namely ALK1) and type II (R-II) cell surface receptors that exhibit serine/threonine kinase activity, as well as to the auxiliary receptor endoglin. Upon ligand binding, the R-II transphosphorylates ALK1 (R-I), which then propagates the signal by phosphorylating receptor-regulated Smads (R-Smads) Smad1/5/8. Once phosphorylated, R-Smads form heteromeric complexes with Smad4 and translocate into the nucleus where they regulate transcriptional activity of target genes, including *Id1*. Endoglin, ALK1, BMP9, and Smad4 proteins are encoded by *ENG*, *ACVRL1*, *GDF2*, and* SMAD4*, whose pathogenic mutations give rise to HHT and JP/HHT, respectively. This figure was adapted from (Fernández-L et al., 2006).

Hereditary hemorrhagic telangiectasia is a genetically heterogeneous disorder caused by mutations in one of multiple genes in the transforming growth factor-beta (TGF-β) signaling pathway that regulates cell proliferation, differentiation, apoptosis, and migration. Endoglin (*ENG*, chromosome 9q34), activin A receptor type II-like 1 (*ACVRL1/ALK1*, chromosome 12q13), and *SMAD4* (chromosome 18q21) mutations cause HHT1 (OMIM 187300), HHT2 (OMIM 600376), and the combined Juvenile Polyposis/HHT (JP/HHT) syndrome (OMIM 175050), respectively (Shovlin et al., 1994; Johnson et al., 1996; Gallione et al., 2004). *ENG* was identified as an HHT causative gene 20 years ago (McAllister et al., 1994) followed by *ACVRL1* in Johnson et al. (1996) and *SMAD4* Gallione et al. (2004). *ENG* and *ACVRL1* encode TGF-β pathway receptor proteins that are involved in the phosphorylation of Smad proteins and regulation of downstream signaling (**Figure 1**).

Endoglin and *ACVRL1* mutations are detected in approximately 85% of cases submitted to clinical molecular genetics laboratories for clinical suspicion of HHT. Over 750 pathogenic *ENG* and *ACVRL1* mutations have been described to date (HHT mutation databases^1^^,^^2^ ). The HHT mutation database^3^ is the most comprehensive database for endoglin and ACVRL1 mutations described in HHT cases and is representative of a collection of mutations described in the literature as well as submissions from clinicians and researchers from around the world. Phenotype information, when available, is listed along with the variant classification (i.e., pathogenic, variant of uncertain significance, or benign) and reference for each genetic variation. Most mutations in *ENG* and *ACVRL1*, which together account for roughly equal percentages of the disorder (Bayrak-Toydemir et al., 2004), lead to the underproduction of their respective proteins and excessive abnormal angiogenesis (Abdalla and Letarte, 2006). The analysis of endoglin and ALK1 protein levels in affected HHT patients strongly supports haploinsufficiency and the associated reduced levels of functional protein as the underlying cause of HHT1 and HHT2, respectively. Missense mutations are the most common mutation type observed in *ENG* and *ACVRL1*, and mutations have been identified in all exons of both genes. Although less common than missense mutations in *ENG* and *ACVRL1*, the proportion of mutations causing a truncating frameshift or stop codon (i.e., indels and non-sense mutations) are more frequent in *ENG* than in *ACVRL1* (Lesca et al., 2004^3^).

No common mutation “hotspots” have been observed in either gene and mutations have been observed across all coding regions. However, many *ENG* mutations have been identified in the extracellular region of the protein, the largest part of the protein (Abdalla and Letarte, 2006). Large deletions or duplications of one or more exons account for 6–10% of all *ENG* and *ACVRL1* mutations (McDonald et al., 2011a). Recently, mutations in the 5′UTR region of *ENG* (c.-9G > A and c.-127C > T) were also shown to cause HHT in several patients, indicating the need for the inclusion of the *ENG* 5′UTR region in routine molecular diagnostic testing for HHT (Damjanovich et al., 2011).

*De novo* mutations and mosaicism involving these two genes are rare, but have both been reported. Two patients with no family history of HHT were shown to be mosaic for a mutation in *ACVRL1* and *ENG*, respectively (Best et al., 2011). In addition, three cases have been confirmed to represent *de novo* mutations in a series of 126 patients known or suspected to have HHT (Gedge et al., 2007). Also a large endoglin deletion was shown to convert from an exon 4–7 deletion to a *de novo* exon 3 deletion in one generation within a family. This was likely due to non-homologous end-joining (NHEJ) repair of a common breakpoint in *ENG* intron 3 (Wooderchak et al., 2010b). This finding has implications for molecular diagnostics since targeted family specific mutation analysis for exon deletions could have led to the misdiagnosis of some affected family members.

A* SMAD4* mutation is detected in less than 2% of cases submitted for clinical suspicion of HHT (Gallione et al., 2006; Lesca et al., 2006; Prigoda et al., 2006). Nearly 100 *SMAD4* missense, non-sense, frameshift, splice site, and large deletion mutations have been described throughout the gene (Wooderchak et al., 2010a). Initial reports of the combined JP/HHT syndrome suggested that mutations in the MH2 domain were in particular responsible for the HHT phenotype. It is now clear that the spectrum of *SMAD4* mutations in patients or families described as having Juvenile Polyposis Syndrome (JPS) alone are almost identical to those with JP/HHT combined (Gallione et al., 2010). Thus all patients with a mutation in the* SMAD4* gene should be considered at risk for the features of both JPS and HHT.

Approximately 15% of individuals suspected by their physician to have HHT have no mutation in *ENG*, *ACVRL1,* or *SMAD4*. Determining the genetic basis of HHT manifestations in patients who do not have a mutation in one these three genes has remained a great challenge for nearly a decade. Linkage analysis identified two additional HHT loci at chromosome 5q31 (Cole et al., 2005) and chromosome 7p14 (Bayrak-Toydemir et al., 2006b), but the genes still remain unknown. One patient with signs of HHT and pulmonary arterial hypertension was found to have a non-sense mutation in the bone morphogenetic protein receptor, type II gene, *BMPR2* (Rigelsky et al., 2008).

Recently, exome sequencing was used to identify missense mutations in the bone morphogenetic 9 (*BMP9)* gene (*GDF2*, chromosome 10q11) in two unrelated individuals suspected to have HHT who previously tested negative for *ENG*, *ACVRL1*, and *SMAD4.* A third case was identified by Sanger sequencing of *GDF2* in additional such individuals (Wooderchak-Donahue et al., 2013). These patients had epistaxis, telangiectasia, and some reported family history of symptoms associated with HHT- primarily epistaxis (OMIM 615506; Wooderchak-Donahue et al., 2013). BMP9 exerts its functional effects by binding to specific endothelial cell surface receptors, namely the auxiliary receptor endoglin and the serine-threonine kinase, ALK1, both members of the highly conserved TGF-β superfamily. The BMP9-dependent activation of ALK1 leads to the phosphorylation of Smad1, Smad5, and Smad8. The resulting phospho-Smad proteins associate with Smad4 to form a Smad complex that translocates to the nucleus to regulate gene expression in human microvascular endothelial cells (Figure 1, adapted from Fernández-L et al., 2006). The overall contribution of* BMP9* mutations for HHT is estimated to be <1% (Wooderchak-Donahue et al., 2013).

## GENOTYPE-PHENOTYPE CORRELATIONS

The common, classic features of HHT: mucocutaneous telangiectasia in similar locations (cluster on lips, mouth, hands, and intestines), epistaxis resulting from nasal telangiectases, and pulmonary, cerebral and hepatic AVMs, are all found in HHT1, HHT2, and JP/HHT. However, the rate of solid organ AVMs is noted to be different between the subtypes. In particular, pulmonary and cerebral AVMs have been shown to be somewhat more common in patients with HHT1 than with HHT2; on the other hand, hepatic manifestations of HHT are more common in patients with HHT2 than HHT1 (Bayrak-Toydemir et al., 2006a; Wain et al., 2014). The rate of AVMs in solid organs is less well known in JP/HHT patients, but the small series reported to date suggest that it is not less than in HHT1 and HHT2 (Gallione et al., 2010; Wain et al., 2014).

Pulmonary arterial hypertension (PAH) is a rare manifestation of HHT. It has been reported mostly in HHT2, but has also been reported in HHT1 (Trembath et al., 2001; Soubrier et al., 2013). One individual with both PAH and significant clinical suspicion for HHT was found to have a clearly deleterious mutation in *BMPR2* (Rigelsky et al., 2008).

The phenotype associated with a mutation in *GDF2* is currently ill-defined. Pathogenic variants in *GDF2* have been described in only three individuals who had been suspected by their referring doctor to have HHT. But review of available clinical details revealed that in one case some of the dermal telangiectases were larger than the pinpoint-pinhead size lesions that typify HHT – more like those described in patients with Capillary Malformation- AVM Disorder (Revencu et al., 2013). Liver imaging in this same patient was suggestive of hepatic vascular findings seen in HHT patients. However, the individual had also been diagnosed with hepatopulmonary syndrome and the cause of her hepatic issues is not clear. In another case, the telangiectases were more numerous and not limited to the hands, mouth, and face (∼80 on one arm), as is typical of HHT (Wooderchak-Donahue et al., 2013).

Although the above described clinical variation between HHT molecular subtypes has been shown, this is not the case for different mutations within the same gene. Data suggest that most disease-causing mutations in *ACVRL1*, *ENG,* and *SMAD4* are null alleles that result in haploinsufficiency. One mutation (*ENG* c.-9G > A) has been seen in multiple affected individuals, one who was homozygous for this mutation, and expression studies showed decreased protein levels, suggesting a hypomorphic allele (Damjanovich et al., 2011). But as a rule, there does not appear to be phenotype variation dependent on the location or mutation type within these genes (Berg et al., 1997; Pece et al., 1997; Gallione et al., 1998).

## GENETIC MODIFIERS

Hereditary hemorrhagic telangiectasia has significant phenotypic variability, wherein the number and location of telangiectases and AVMs vary widely between individuals even within the same family. The genetic heterogeneity of HHT does not explain the variable clinical symptoms and manifestations routinely seen within families. The significant intra-familial variation in phenotype suggests effects of genetic modifiers on the HHT phenotype. Two genes,* PTPN14* and *ADAM17*, were recently identified as genetic modifiers of angiogenesis and HHT (Benzinou et al., 2012; Kawasaki et al., 2014). *PTPN14* variants have been shown to influence the clinical severity of HHT by being associated with the development of pulmonary AVMs in HHT patients (Benzinou et al., 2012). *ADAM17* variants have been associated with the presence of pulmonary AVMs in HHT1 but not HHT2 and can potentiate a TGF-β regulated vascular disease. Additional, yet undiscovered genetic modifiers may also play a role in the development of vascular lesions seen in HHT.

## MOLECULAR DIAGNOSTICS

### MEDICAL INDICATIONS

Molecular genetic testing to establish the molecular subtype of HHT is recommended when HHT is diagnosed or suspected on clinical grounds. Whether or not the family has a mutation in *SMAD4,* in particular, changes clinical management due to the associated risk of gastrointestinal polyps and malignancy not associated with mutations in other HHT genes. While mutations in *SMAD4* cause only a small percentage (∼2%) of all HHT (Gallione et al., 2006; Lesca et al., 2006; Prigoda et al., 2006), the medical management considerations for the resulting JP/HHT Syndrome are such that ruling out a mutation in this gene is clinically relevant. In practice a *SMAD4* mutation is usually “ruled out” in an HHT family proband by the detection of a disease causing *ACVRL1* or *ENG* mutation, rather than “ruled in” by analysis of the *SMAD4* gene itself.

Secondly, if a pathogenic gene mutation is identified in an affected family proband, early diagnostic testing is then available as recommended for at-risk family members. In contrast to many hereditary disorders, diagnostic genetic testing for HHT is recommended early in life for first degree relatives of affected individuals, even when asymptomatic or minimally symptomatic. The diagnosis of HHT can rarely be made, and can never be ruled out, based purely on medical history and physical examination in the first decade of life. This is because the average age of epistaxis onset is 12 years, and multiple mucocutaneous telangiectases are often not apparent until the third decade (Assar, 1991; Porteous et al., 1992); yet solid organ AVMs are usually present from birth. Because serious complications of pulmonary and cerebral AVMs can occur without warning at an early age, and most are preventable, (Morgan et al., 2002; Mei-Zahav et al., 2006), screening for pulmonary and cerebral AVMS is recommended in all affected children (Al-Saleh et al., 2009; Faughnan et al., 2011). HHT is a disorder in which recommended medical management is changed by making an early diagnosis.

### HHT MOLECULAR DIAGNOSTICS TO DATE

The genetic complexity and number of mutations described in HHT have made it challenging to determine an efficacious algorithm for diagnostic testing. Since molecular diagnostic testing became available a decade ago, the norm has been an algorithm that involved Sanger sequencing for point mutations and multiplex ligation-dependent probe amplification (MLPA) for the detection of large deletions and duplications in *ENG*, *ACVRL1*, and* SMAD4*. The results of 383 consecutive samples submitted to ARUP Laboratories (2014a,b) for HHT molecular testing between 2007 and 2009 were reviewed and led our group to propose a testing algorithm in which the *ENG* and *ACVRL1* genes were simultaneously sequenced and analyzed for large deletions and duplications as the initial analysis. This approach accounted for the fact that the majority of these 383 family probands had required both genes to be fully analyzed for optimal mutation detection and accurate interpretation of results. When a unique missense mutation was (frequently) detected in either *ACVRL1* or *ENG* gene, it was helpful to know that no deletion/duplication was detected in the same gene; and no mutation by either sequencing or deletion/duplication analysis in the other gene. In addition, since *ACVRL1* and *ENG* each account for slightly less than half of all cases, the need to test the other gene in a particular case was the rule. In our experience, when efficiency (related to cost), accuracy and turnaround time were considered, a protocol by which *ENG* and *ACVRL1* were simultaneously analyzed, with reflex to *SMAD4* if both were negative, has seemed the most efficacious algorithm for molecular diagnostics for most of the last decade (McDonald et al., 2009, 2011b).

### CLINICAL SENSITIVITY

To date, reports of the detection rate for a mutation in *ENG* and *ACVRL1* in HHT have come from laboratories, which receive samples from clinicians with a wide range of expertise with regards to recognizing clinical manifestations of HHT (Richards-Yutz et al., 2010; McDonald et al., 2011b). In general, a mutation is detected in either *ENG* or *ACVRL1* in approximately 75% of cases in which the ordering physician reports the individual to have HHT. But Richards-Yutz et al. (2010) reported that the mutation detection rate for *ENG* and *ACVRL1* ranged from 85% if the ordering physician specifically reported the patient to have all four Curaçao diagnostic criteria (Epistaxis, Telangiectases, AVM, Family History); to ∼40% if three diagnostic criteria were reported, *and* the three criteria were specifically telangiectasia, AVM and family history. It is clear that all clinical manifestations and all physician assessments are not equally predictive of having a mutation in *ENG* or *ACVRL1*, the two common genes for HHT.

Our group forms the core of the University of Utah HHT Center, which has provided clinical diagnosis and medical management for this disorder since 1995. We recently reviewed our patient database for individuals who upon physical examination, medical history and family history met clinical diagnostic criteria based on a strict application of the Curaçao criteria (IRB 00039582). This meant three or more of the following: multiple mucocutaneous telangiectases in *characteristic* locations; recurrent and *ongoing* epistaxis; pulmonary, cerebral, hepatic, and/or spinal AVM(s); and a first degree relative who meets diagnostic criteria. 95.7% of such individuals/family probands had a mutation in *ENG* or *ACVRL1* that was known or suspected to be pathogenic; 1.4% in *SMAD4*. (McDonald, unpublished data). It is of note that based on mutation prediction programs and family co-segregation studies performed for uncertain variants in these genes over time, it is our experience that most missense mutations initially interpreted as variants of uncertain significance due to insufficient of evidence of causation are actually pathogenic (Bayrak-Toydemir et al., 2008).

Finally, the inclusion of the *ENG* 5′UTR region in HHT molecular diagnostic testing has increased the overall clinical sensitivity (Damjanovich et al., 2011; ARUP, unpublished results). Deep intronic *ENG* and* ACVRL1* mutations can cause HHT by causing splicing defects. In fact, we hypothesize that most of the ∼3% of patients with HHT according to Curaçao criteria who are not found to have a mutation in *ACVRL1, ENG,* or *SMAD4*, have an undetected deep intronic mutation in *ACVRL1* or *ENG*.

### IMPORTANT RECENT ADVANCES IN MOLECULAR GENETIC ANALYSIS

The last several years have seen the transition of whole exome and whole genome sequencing (e.g., next generation sequencing (NGS) or massively parallel sequencing) from use only in research, to use in clinical diagnostics. As is often the case with technology, this transition to wider use is largely related to drastic reductions in cost. NGS allows for many human genes to be sequenced in one orderable test; as opposed to single-gene analysis by Sanger sequencing which has been the mainstay of molecular diagnostics for the last several decades. And NGS testing has been shown to have a higher analytical sensitivity than traditional Sanger sequencing due to increased potential to detect low level mosaicism.

On the other hand, this new technology still adds cost in most cases, and can decrease detection rates for mutations in certain genes. It can also increase turnaround time from weeks to months. Most concerning to many is the increased complexity and potential ambiguity of results. The risk of detecting genetic variants of uncertain clinical significance exists for gene sequencing using any technology. But the chance of detecting a variant of unknown significance, with the inherent uncertainty and anxiety this causes for clinician and patient alike, multiplies relative to how many genes are sequenced. This must be balanced with the advantages of testing multiple genes with one assay.

### NEW CONSIDERATIONS AND PROPOSED TESTING ALGORITHM

An expanded approach to molecular diagnostics for HHT is evolving due to the increasing use of NGS in the clinical molecular diagnostic setting, and the discovery in 2013 of pathogenic mutations in the *GDF2* gene in three patients clinically suspected to have HHT. Also, *RASA1* gene mutations have been detected in a small number of patients who had been clinically suspected to have HHT (Wooderchak-Donahue, unpublished results). Although the appearance and location of the cutaneous capillary malformations or telangiectases described in *RASA1*-related disorder/Capillary Malformation-AVM Syndrome (CM-AVM) are distinguishable from those in HHT, there is enough similarity that making the distinction can be difficult for clinicians without specific expertise in the identification of vascular malformations.

Thus, when the goal is to rule out a mutation in genes associated with the HHT phenotype, there are now arguably *three* genes *SMAD4*, *GDF2,* and* RASA1*, each low yield for a mutation that might reasonably be considered. Yet, a mutation in *ACVRL1* or *ENG* is found in approximately 96% of patients who meet Curaçao clinical diagnostic criteria when strictly applied.

**FIGURE 2:**
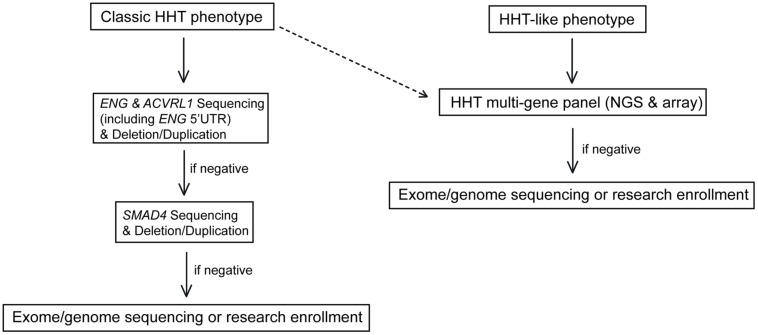
**Proposed molecular testing algorithm for HHT based on the suspected clinical diagnosis.** The testing algorithm for classic HHT is shown on the left. An algorithm for patients suspected to have HHT or an HHT-like phenotype (right) depicts the use of a five gene HHT next generation sequencing panel and aCGH. The dashed line indicates a streamlined future diagnostic algorithm for HHT patients as NGS costs and turn-around times are reduced.

Given the above considerations, we now choose one of two different molecular diagnostic approaches for a family proband who presents in our specialty clinic for HHT (**Figure 2**). If evaluation based on the targeted medical history, physical examination, and multi-generation family history suggests:

(1) Classic HHT as defined by the Curaçao criteria; testing of *ENG* and *ACVRL1* by Sanger Sequencing and deletion/duplication analysis with reflex to *SMAD4* if *ENG* and *ACVRL1* test results are negative.
(2) Suspicion for a hereditary telangiectasia syndrome but not necessarily for classic HHT (e.g., telangiectases with atypical distribution, lack of epistaxis, lack of solid organ involvement); a five gene (*ENG, ACVRL1, SMAD4, RASA1,* and *GDF2*) NGS panel (**Table 1**) and corresponding array comparative genomic hybridization (aCGH) for the detection of large deletions and duplications in the same five genes.

**Table 1:** Hereditary hemorrhagic telangiectasia next generation sequencing panel genes.

Gene name	Chromosomal locus	Protein name	Disease	Reference sequence	Exons
*ENG*	9q33-q34.1	Endoglin	HHT1	NM_001114753	14
*ACVRL1*	12q11-q14	Activin A receptor type II-like 1 (ALK1)	HHT2	NM_000020	10
*SMAD4*	18q21.1	SMAD, mothers against DPP homolog 4 (*Drosophila*) MADH4	JPS/HHT	NM_005359	12
*GDF2/BMP9*	10q11.22	growth differentiation factor 2	HHT-like	NM_016204	2
*RASA1*	5q13.3	RAS p21 protein activator (GTPase activating protein) 1	RASA1-related disorders CM/AVM Parkes Weber Syndrome	NM_002890	25

*This five gene HHT panel is available for clinical testing at ARUP Laboratories. HHT, hereditary hemorrhagic telangiectasia; JPS, juvenile polyposis syndrome*.

If a causative mutation is not identified by the chosen approach, symptom guided exome sequencing is considered (**Figure 2**).

## CONCLUSION AND FUTURE DIRECTIONS

With the advent of NGS techniques, a new era for gene discovery, genetic modifier identification to explain the clinical variability seen in HHT, and molecular diagnostics for HHT has begun. The discovery last year that a mutation in *GDF2* can cause a phenotype similar to HHT was made possible by exome sequencing. The addition of this gene to the growing list of genes to be considered when a patient is suspected to have a hereditary telangiectasia syndrome, in turn increases the potential efficacy of a multiple gene panel test made possible by NGS when deciding on a molecular diagnostic test.

Additional genes associated with an HHT phenotype will likely be identified. By definition, each will be a small contributor to the overall incidence of HHT. But additional genes can easily be added to an HHT NGS panel to increase clinical sensitivity. The more genes that are identified, and as the cost and turn-around time for NGS and complementary aCGH continue to go down with time, this assay will likely become the method of choice for the molecular diagnosis of individuals suspected to have HHT (dashed line, **Figure 2**). Also, the contribution of deep intronic, UTR, or promoter variants of one of the known genes (*ENG*, *ACVRL1*, or *SMAD4*) is still not well known. As the non-coding and regulatory regions of these genes are better understood, these regions can also be included in diagnostic testing to increase the overall clinical sensitivity of HHT molecular diagnostics.

Importantly, HHT patients who had molecular genetic testing in the past, in whom no pathogenic mutation was detected, should be considered at the time of follow-up consultation for retesting or additional testing using current protocols and techniques. Molecular diagnostics 10 years ago involved only sequencing of coding regions of* ENG* and *ACVRL1*. Large deletions and duplications in these genes would have been missed as would mutations in the more recently discovered genes associated with an HHT phenotype. Also, mutations in the UTR region of ENG would not have been detected.

It is increasingly apparent that vascular lesions in other inherited vascular malformation syndromes often harbor a somatic second genetic hit mutation (Akers et al., 2009; Limaye et al., 2009; Amyere et al., 2013; Revencu et al., 2013). Biallelic germline and somatic mutations were identified in cerebral cavernous malformations (CCMs) in all three forms of inherited CCM, suggesting that the formation of these lesions requires complete loss of function of a CCM gene (Akers et al., 2009). A somatic second hit mutation was also identified in tissue from a capillary malformation in a patient with a germline *RASA1* mutation (Revencu et al., 2013). In addition, inherited venous malformations caused by *GLMN* and *TIE2* mutations were also shown to follow the somatic second hit model (Limaye et al., 2009; Amyere et al., 2013). It will be interesting to determine whether telangiectasias and AVMs from HHT patients also have a somatic second hit mutation. This may help explain the presence of multiple vascular lesions in the same individual and the variable phenotypes observed in families who carry the same mutation.

Overall, it has become apparent that the genetics as well as phenotype of HHT are more complex than known and thus described when the Curaçao diagnostic criteria for HHT were set forth (Shovlin et al., 2000). At that time in 2000, only the* ENG* and *ACVRL1* genes were known to be associated with HHT and Curaçao clinical diagnostic criteria have stood the test of time for describing the syndromes caused by mutations in these two genes. Since then, however, two additional genes have been discovered, mutations that cause a hereditary telangiectasia syndrome which does not clearly fit this definition of HHT. When our group reported last year that mutations in *GDF2* were found in three patients with a telangiectasia syndrome, a decision was made to stop short of labeling it a new type of HHT, because it was not clear that the dermal telangiectases were typical of HHT as described in the Curaçao criteria. Also, solid organ AVMs were not confirmed in any of the three patients, although this could be attributable to the small number of individuals who had not been thoroughly evaluated (Wooderchak-Donahue et al., 2013). We have observed in our clinical center for HHT a number of families that are compelling for having some type of hereditary telangiectasia disorder, yet do not have a mutation in one of the currently known genes (*ENG, ACVRL1, SMAD4,* and* GDF2*).

Future molecular characterization of families referred for suspicion of having HHT will likely require either an expanded definition of HHT itself, or an expanded categorization of telangiectasia/AVM disorders which includes HHT. But it is clear that in the near future molecular diagnostic NGS panel assays and exome and genome sequencing will help elucidate additional genetic aberrations – both primary causative genes and modifiers of phenotype- in HHT patients that have remained elusive for years.

## Conflict of Interest Statement

The authors declare that the research was conducted in the absence of any commercial or financial relationships that could be construed as a potential conflict of interest.

## Footnotes

1 http://www.arup.utah.edu/database/ACVRL1/ACVRL1_welcome.php

2 http://www.arup.utah.edu/database/ENG/ENG_welcome.php

3 http://www.arup.utah.edu/database/hht/
